# Supplementary material for: Inhibition of CBP synergizes with the RNA-dependent mechanisms of Azacitidine by limiting protein synthesis
Source: Nat Commun. 2021 Oct 18;12:6060. doi: 10.1038/s41467-021-26258-z (PMC8523560; doi:10.1038/s41467-021-26258-z)
Supplement: Supplementary file 1 — Supplementary Information [file 41467_2021_26258_MOESM1_ESM.pdf]

Supplementary Information

Inhibition of CBP synergizes with the RNA-dependent mechanisms of Azacitidine by limiting protein synthesis

Short title: CBP inhibition synergizes with AZA

Jeannine Diesch[1,2], Marguerite-Marie Le Pannérer[1], René Winkler[1], Raquel Casquero[1], Matthias Muhar[3], Mark van der Garde[4,5], Michael Maher[1], Carolina Martínez Herráez[6,7], Joan J. Bech-Serra[1], Michaela Fellner[3], Philipp Rathert[3,11], Nigel Brooks[8], Lurdes Zamora[9], Antonio Gentilella[6,7], Carolina de la Torre[1], Johannes Zuber[3,10], Katharina S. Götze[4], Marcus Buschbeck[1,2,#]

1 Cancer and Leukaemia Epigenetics and Biology Program, Josep Carreras Leukaemia Research Institute (IJC), Campus ICO-GTP-UAB, Badalona, Spain

2 Program for Predictive and Personalized Medicine of Cancer, Germans Trias i Pujol Research Institute (PMPPC-IGTP), Badalona, Spain

3 Research Institute of Molecular Pathology (IMP), Vienna BioCenter (VBC), Vienna, Austria.

4 Department of Medicine III, Klinikum rechts der Isar, Technische Universität München, Munich, Germany.

5 German Cancer Consortium (DKTK), Heidelberg, Partner Site Munich, Germany.

6 Laboratory of Cancer Metabolism, ONCOBELL Program, Institut d'Investigació Biomèdica de Bellvitge (IDIBELL), L'Hospitalet de Llobregat, Spain.

7 Faculty of Pharmacy, Department of Biochemistry and Physiology, Universitat de Barcelona, Barcelona, Spain

8 CellCentric, Ltd, Chesterford Research Park, Little Chesterford, Cambridge, CB10 1XL, UK.

9 Hematology Laboratory Service, ICO Badalona-Hospital Germans Trias I Pujol, Josep Carreras Leukemia Research Institute (IJC), Badalona, Spain.

10 Medical University of Vienna, Vienna BioCenter (VBC), Vienna, Austria.

11 Present: Biochemistry Department, University Stuttgart, Stuttgart, Germany.

[#]Correspondence to: Marcus Buschbeck, email: mbuschbeck@carrerasresearch.org

Supplementary Figure S1

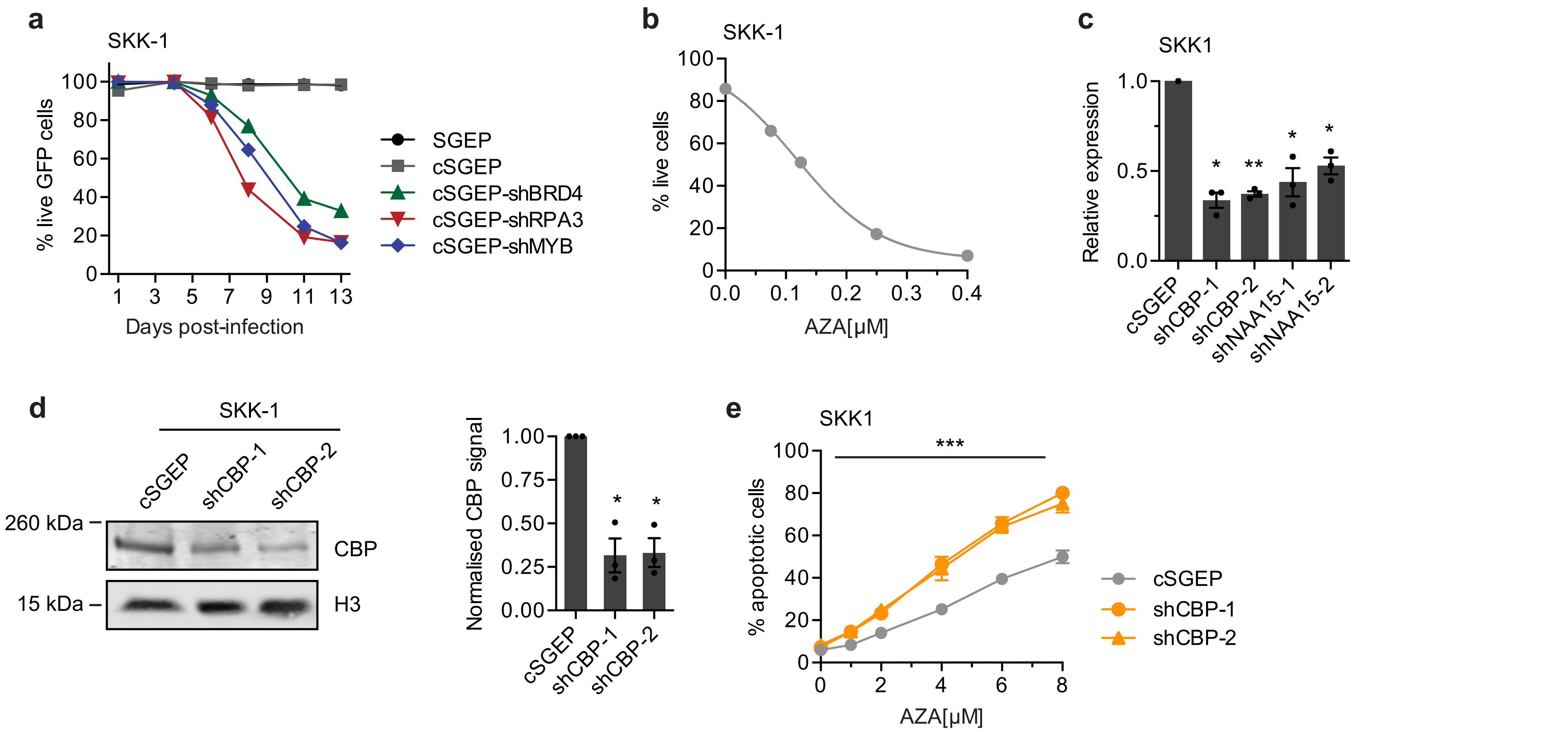

Figure S1. Optimization of shRNA screen and generation of stable knockdown cells. (a) Percentage of GFP-positive SKK-1 cells stably expressing SGEP, cSGEP, shBRD4, shRPA3 or shMYB determined at different time points post-infection. (b) Percentage of live SKK-1 cells determined after 14 days of treatment with different concentrations of AZA (every 2 days). Data represent the mean ±SEM of two independent experiments. (c) Knockdown efficiency of two different shRNAs against CBP and NAA15 was measured by RT-qPCR and control cells with cSGEP were set to 1. Data represent the mean ±SEM of three independent measurements. shCBP-1, *P*=0.004; shCBP-2, *P*=0.0005; shNAA15-1, *P*=0.019, shNAA15-2, *P*=0.0098. (d) Western blot of expression of CBP and H3 as loading control in SKK-1 cells stably expressing cSGEP, shCBP-1 or shCBP-2. The signal was quantified using the Odyssey software and is represented as the mean ±SEM of three independent measurements. shCBP-1, *P*=0.0198; shCBP-2, *P*=0.015. (e) SKK-1 cells stably expressing cSGEP, shCBP-1 or shCBP-2 were treated for 4 days with indicated concentrations of AZA followed by measurement of the percentage of apoptotic cells indicated AnnexinV staining. Data represent the mean ±SEM of three independent experiments. Statistical analysis was performed using two-way ANOVA. ***, p-value < 0.0001. (c-d) Statistical analysis was performed by two-sided Student's T-test compared to untreated samples, *p-value < 0.05, **p-value < 0.001. Source data are provided as a Source Data file.

Supplementary Figure S2

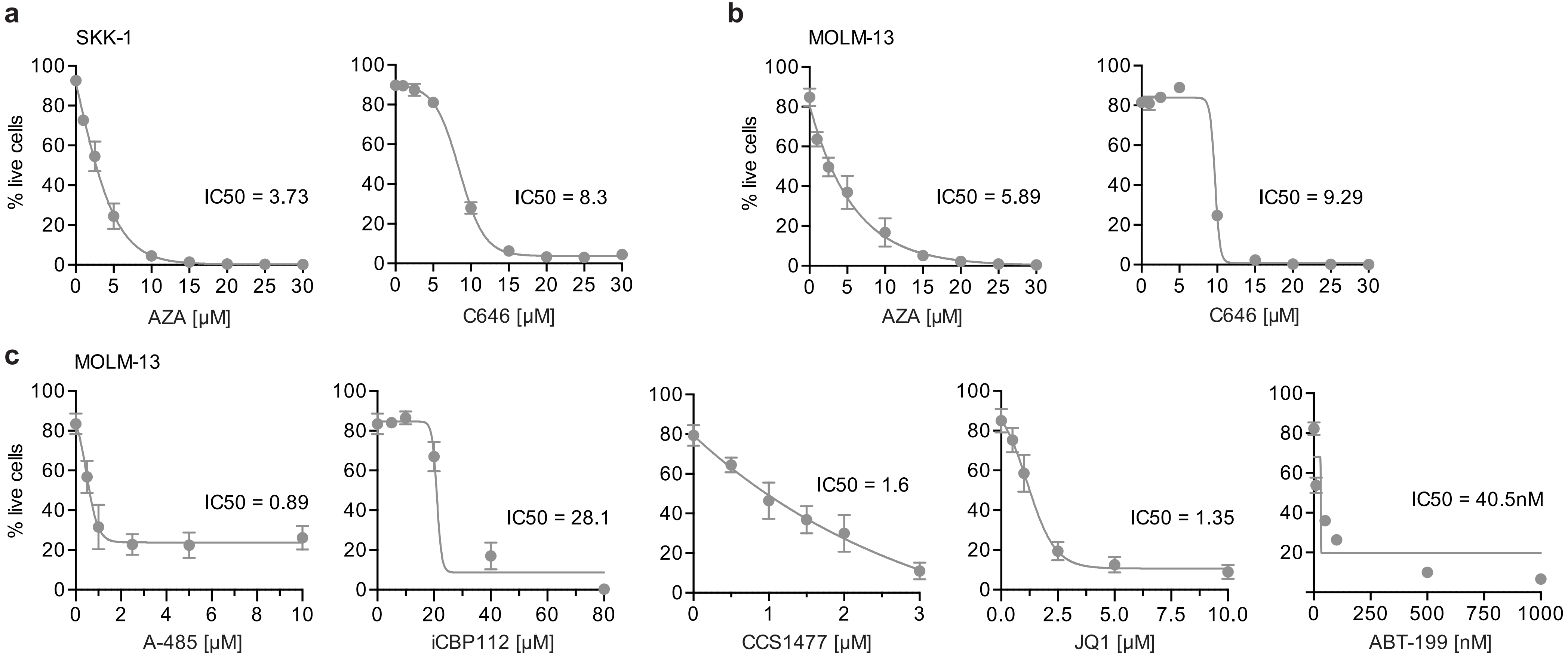

Figure S2. IC50 calcuations in SKK-1 and MOLM-13 cells. (a) Dose response curves in SKK-1 cells were determined after 4 days of treatment with different concentrations of AZA (0, 1, 2.5, 5, 10, 15, 20, 25, 30 µM) or C646 (0, 1, 2.5, 5, 10, 15, 20, 25, 30 µM). (b) Dose response curves in MOLM-13 cells were determined by DAPI/MitoTracker staining after 4 days of treatment with different concentrations of AZA (0, 1, 2.5, 5, 10, 15, 20, 25, 30 µM) or C646 (0, 1, 2.5, 5, 10, 15, 20, 25, 30 µM). (c) Dose response curves in MOLM-13 cells as determined by DAPI/MitoTracker staining after 4 days of treatment with different concentrations of A-485 (0, 0.5, 1, 2.5, 5, 10 µM), iCBP-112 (0, 5, 10, 20, 40, 80 µM), CCS1477 (0, 0.5, 1, 1.5, 2, 3 µM), JQ-1 (0, 0.5, 1, 2.5, 5, 10 µM) or ABT-199 (0, 10, 50, 100, 500, 1000 nM). Data represent the mean ±SEM of three independent experiments. IC50 values were calculated using Graphpad prism. Source data are provided as a Source Data file.

Supplementary Figure S3

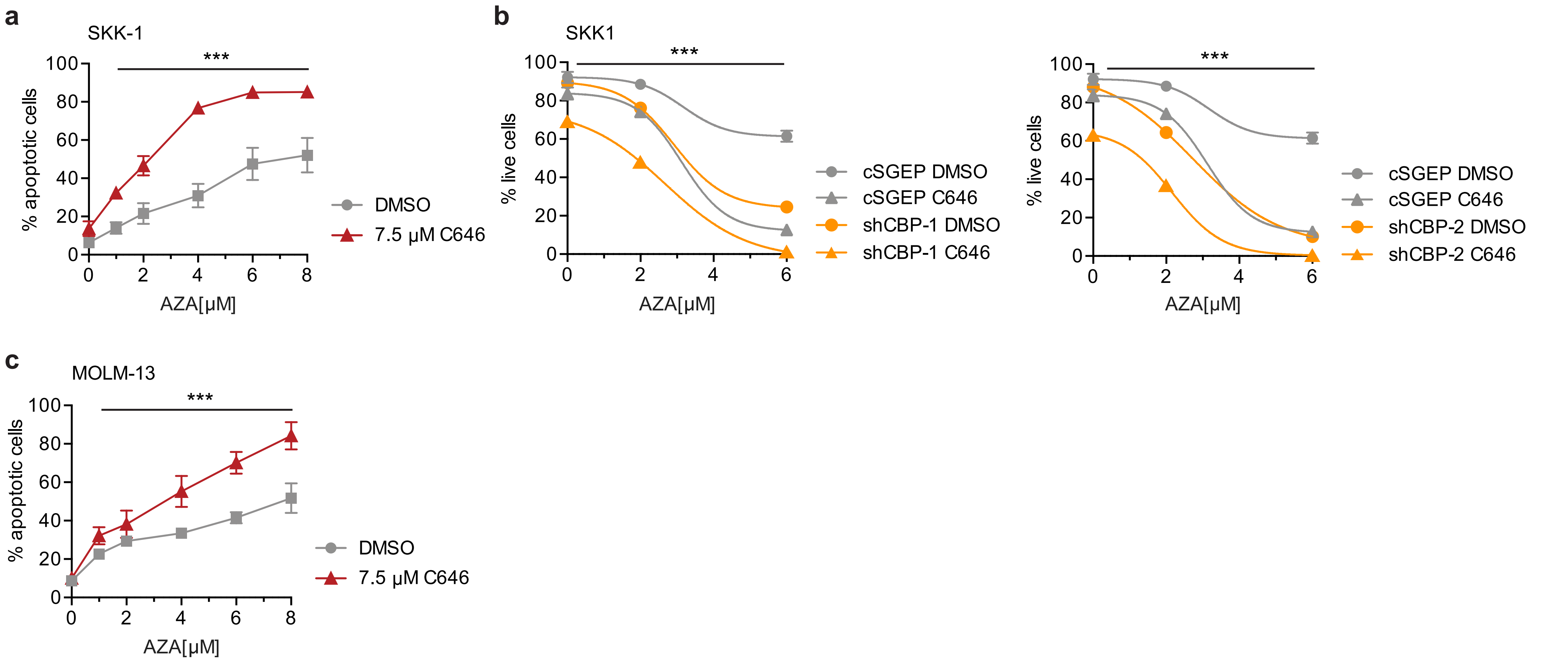

Figure S3. Apoptosis assay in SKK-1 and MOLM-13. (a) Percentage of apoptotic SKK-1 assessed by AnnexinV staining after 4 days of treatment with indicated concentrations of AZA in combination with C646 or 0.075% DMSO as vehicle control. (b) SKK-1 cells stably expressing cSGEP, shCBP-1 or shCBP-2 were treated for 4 days with indicated concentrations of AZA and 7.5 μM C646 or 0.075% DMSO followed by measurement of the percentage of live cells. (c) Percentage of apoptotic MOLM-13 assessed by AnnexinV staining after 4 days of treatment with indicated concentrations of AZA in combination with C646 or 0.075% DMSO as vehicle control. (a-c) Data represent the mean ±SEM of at least three independent experiments. Statistical analysis was performed using two-way ANOVA. ***, p-value < 0.0001. Source data are provided as a Source Data file.

Supplementary Figure S4

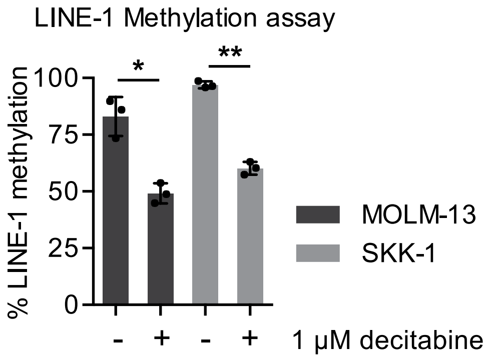

Figure S4. Validation of decitabine treatment and protein synthesis in CBP knockdown cells. LINE-1 methylation was determined in MOLM-13 and SKK-1 cells after 24 hours incubation if 1 µM decitabine using the LINE-1 methylation assay (active motif). Data represent the mean ±SEM of three independent experiments. Statistical analysis was performed by two-sided Student's T-test compared to untreated samples, MOLM-13, P=0.02; SKK-1, P=0.0006. *P-value < 0.05; **P-value < 0.001. Source data are provided as a Source Data file.

Supplementary Figure S5

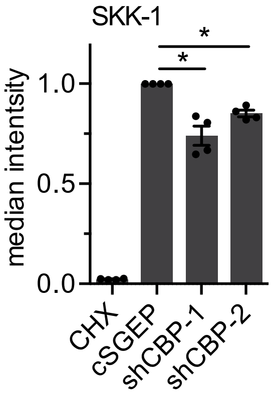

Figure S5. Protein synthesis in CBP knockdown cells. Median fluorescence in SKK-1 cells stably expressing cSGEP, shCBP-1 or shCBP-2 by Click-iT™ HPG Alexa Fluor™ 594 assay. SKK-1 cSGEP cells treated 1.5 hours with cycloheximide (CHX) were used as positive control. Data represent the mean ±SEM of four independent experiments. Statistical analysis was performed by two-sided Student's T-test compared to cSGEP untreated control. shCBP-1, *P*=0.012; shCBP-2, *P*=0.0027. *p-value < 0.05. Source data are provided as a Source Data file.

Supplementary Figure S6

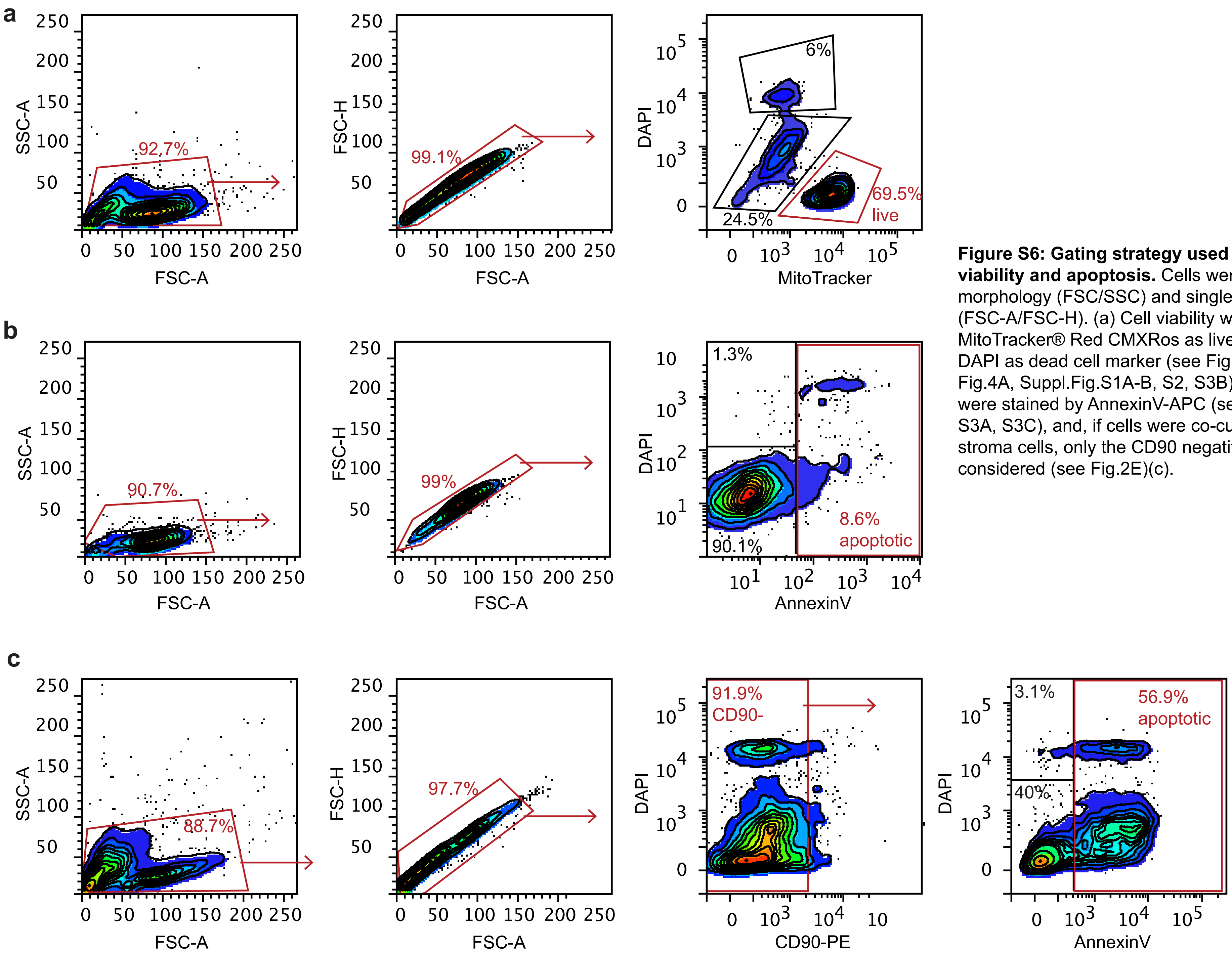

Figure S6: Gating strategy used to analyse cell viability and apoptosis. Cells were gated on cell morphology (FSC/SSC) and single events (FSC-A/FSC-H). (a) Cell viability was determined by MitoTracker® Red CMXRos as live cell marker and DAPI as dead cell marker (see Fig.1F-H, Fig.2A-D, Fig.4A, Suppl.Fig.S1A-B, S2, S3B). (b) Apoptotic cells were stained by AnnexinV-APC (see Suppl.Fig.S1E, S3A, S3C), and, if cells were co-cultured with HS-5 stroma cells, only the CD90 negative fraction was considered (see Fig.2E)(c).
