# Supplementary material for: Inhibition of CBP synergizes with the RNA-dependent mechanisms of Azacitidine by limiting protein synthesis
Source: Nat Commun. 2021 Oct 18;12:6060. doi: 10.1038/s41467-021-26258-z (PMC8523560; doi:10.1038/s41467-021-26258-z)
Supplement: Supplementary file 3 — Description of additional Supplementary File [file 41467_2021_26258_MOESM3_ESM.pdf]

Description of Additional Supplementary Files

File Name: Supplementary Data 1
Description: hEPI9 shRNA library composition.

File Name: Supplementary Data 2
Description: Supplementary methods information: shRNA sequences / qRT-PCR primers / Patient characteristics.

File Name: Supplementary Data 3
Description: hEPI9 shRNA screen results in SKK-1 cells after 14 days of treatment with AZA.

File Name: Supplementary Data 4
Description: Differentially expressed genes determined by SLAM-seq in MOLM-13 cells after 2 hours of treatment with C646.

File Name: Supplementary Data 5
Description: GO analysis of down- or up-regulated newly synthesized transcripts in MOLM-13 cells after 2 hours of treatment with C646.

File Name: Supplementary Data 6
Description: Newly synthesized proteins in MOLM-13 cells after 4 hours of treatment with C646.

File Name: Supplementary Data 7
Description: GO analysis of affected proteins with $\geq$ 75 % decrease in PSM in MOLM-13 cells after 4 hours of treatment with C646.

File Name: Supplementary Data 8
Description: Polysome profiling in MOLM-13 cells after 4 hours of treatment with C646.
