# Supplementary material for: Inhibition of CBP synergizes with the RNA-dependent mechanisms of Azacitidine by limiting protein synthesis
Source: Nat Commun. 2021 Oct 18;12:6060. doi: 10.1038/s41467-021-26258-z (PMC8523560; doi:10.1038/s41467-021-26258-z)
Supplement: Supplementary file 12 — Reporting Summary [file 41467_2021_26258_MOESM12_ESM.pdf]

nature research

Corresponding author(s): Marcus Buschbeck

Last updated by author(s): Sep 10, 2021

Reporting Summary

Nature Research wishes to improve the reproducibility of the work that we publish. This form provides structure for consistency and transparency in reporting. For further information on Nature Research policies, see our Editorial Policies and the Editorial Policy Checklist.

Statistics

For all statistical analyses, confirm that the following items are present in the figure legend, table legend, main text, or Methods section.

n/a	Confirmed	
☐	☒	The exact sample size (*n*) for each experimental group/condition, given as a discrete number and unit of measurement
☐	☒	A statement on whether measurements were taken from distinct samples or whether the same sample was measured repeatedly
☐	☒	The statistical test(s) used AND whether they are one- or two-sided<br>*Only common tests should be described solely by name; describe more complex techniques in the Methods section.*
☐	☒	A description of all covariates tested
☐	☒	A description of any assumptions or corrections, such as tests of normality and adjustment for multiple comparisons
☐	☒	A full description of the statistical parameters including central tendency (e.g. means) or other basic estimates (e.g. regression coefficient) AND variation (e.g. standard deviation) or associated estimates of uncertainty (e.g. confidence intervals)
☐	☒	For null hypothesis testing, the test statistic (e.g. *F*, *t*, *r*) with confidence intervals, effect sizes, degrees of freedom and *P* value noted<br>*Give P values as exact values whenever suitable.*
☒	☐	For Bayesian analysis, information on the choice of priors and Markov chain Monte Carlo settings
☒	☐	For hierarchical and complex designs, identification of the appropriate level for tests and full reporting of outcomes
☒	☐	Estimates of effect sizes (e.g. Cohen's *d*, Pearson's *r*), indicating how they were calculated

Our web collection on statistics for biologists contains articles on many of the points above.

Software and code

Policy information about availability of computer code

Data collection	All data in this study was collected via commercial software as described in detail in the appropriate Methods sections and includes the Applied BiosystemsTM QuantStudioTM 7 Flex Real-Time PCR System for real-time quantitative PCR, the Illumina HiSeq2500 platform for all NGS, the BD FACSDivaTM software for flow cytometry, the Xcalibur software vs 4.0.27.10 for Mass spectometry and BIOCOMP gradient station for polysome profiling. Any additional detail required will be provided on request and prior to publication.
Data analysis	All data in this study is described in detail in the appropriate Methods sections and were analysed via commercial code and/or R-packages, including: GraphPad Prism software (version 6) for statistical analysis; SLAM-Dunk v0.4.1 and DEseq2 package version 1.26.0 for SLAM-seq; DAVID 6.8 gene ontology analysis; MaxQuant software 1.6.7.0 for Mass spectometry; EdgeR for shRNA screen analysis; BD FACSDivaTM software, FlowJo v10 and FlowLogic v8 for flow cytometry, CompuSyn software (version 1) to calculate the combination index.<br>Any additional detail required will be provided on request and prior to publication.

For manuscripts utilizing custom algorithms or software that are central to the research but not yet described in published literature, software must be made available to editors and reviewers. We strongly encourage code deposition in a community repository (e.g. GitHub). See the Nature Research guidelines for submitting code & software for further information.

Data

Policy information about availability of data

All manuscripts must include a data availability statement. This statement should provide the following information, where applicable:

- Accession codes, unique identifiers, or web links for publicly available datasets
- A list of figures that have associated raw data
- A description of any restrictions on data availability

Data from the shRNA screen and SLAM-Seq data are available at GEO under the accession number GSE158738 and GSE158813, respectively. Proteomics data are

available at PRIDE under the accession number PXD021738.
• GSE158738: https://www.ncbi.nlm.nih.gov/geo/query/acc.cgi?acc=GSE158738
Password: cjoxmeuqllcplgn
• GSE158813: https://www.ncbi.nlm.nih.gov/geo/query/acc.cgi?acc=GSE158813
Password: clarccecnrgjfav
• PXD021738: https://
www.ebi.ac.uk/pride/archive
Username: reviewer_pxd021738@ebi.ac.uk
Password: KyQAnegR
For protein alignment the Swissprot/Uniprot human database was downloaded from the www.uniprot.org web site (October, 2019).
For SLAM-seq alignment, the human genome reference GRCh38 was downloaded from https://www.ncbi.nlm.nih.gov/

Field-specific reporting

Please select the one below that is the best fit for your research. If you are not sure, read the appropriate sections before making your selection.

☒ Life sciences ☐ Behavioural & social sciences ☐ Ecological, evolutionary & environmental sciences

For a reference copy of the document with all sections, see nature.com/documents/nr-reporting-summary-flat.pdf

Life sciences study design

All studies must disclose on these points even when the disclosure is negative.

Sample size	No sample-size calculations were performed. Sample size was considered adequate based on the consistency of the results. In most experiments at least 3 biological replicates were used as indicated in the relevant Method sections and Figure legends.
Data exclusions	No data were excluded from any analyses.
Replication	We have performed at least 3 independent biological replicates for each experiment
Randomization	Samples were randomly assigned to experimental groups
Blinding	Blinding was not relevant for this study because no bias could be made by the person performing the experiments.

Reporting for specific materials, systems and methods

We require information from authors about some types of materials, experimental systems and methods used in many studies. Here, indicate whether each material, system or method listed is relevant to your study. If you are not sure if a list item applies to your research, read the appropriate section before selecting a response.

Materials & experimental systems

n/a	Involved in the study	
☐	☒	Antibodies
☐	☒	Eukaryotic cell lines
☒	☐	Palaeontology and archaeology
☒	☐	Animals and other organisms
☐	☒	Human research participants
☒	☐	Clinical data
☒	☐	Dual use research of concern

Methods

n/a	Involved in the study	
☒	☐	ChIP-seq
☐	☒	Flow cytometry
☒	☐	MRI-based neuroimaging

Antibodies

Antibodies used	anti-CREBBP (Abcam, Cambridge, UK, ab2832), anti-histone H3 (Abcam, ab1791), BD Pharmingen™ PE Mouse Anti-Human CD90 Clone: 5E10 (BD Biosciences, Cat: 561970)
Validation	anti-CREBBP has been validated by Abcam by immunoblotting in HeLa cell lysates, immunofluorescent analysis in MCF-7 and NIH-3T3 cells, immunohistochemical analysis in FFPE mouse colon tissue and FFPE human pancreas tissue. anti-histone H3 has been validated by Abcam by immunoblotting in A431, Jurkat, HEK293, NIH-3T3, Drosophila embryo, S.cerevisiae and S.pombe cell lysates, by chromatin immunoprecipitation in HeLa cell extracts, immunofluorescent analysis in HeLa cells, immunohistochemical analysis in rat brain tissue. anti-human CD90 has been validated in the following publications: PMID 1372992, PMID 7683034, PMCID PMC1384357. Further information can be found on the manufacturers website.

Eukaryotic cell lines

Policy information about cell lines

Cell line source(s)	MOLM-13 (DMSZ # ACC554), F36-P (DMSZ # ACC543) and SKK-1 have been obtained from DSMZ as a collaboration with Hans Drexler. MDS-L cells were kindly provided by Kaoru Tohyama. HEK293T (ATCC # CRL-11268) and HS-5 (ATCC # CRL-11882) were obtained from ATCC.
Authentication	Authentication by STR profiling
Mycoplasma contamination	All cell lines are tested once a month and are mycoplasma negative
Commonly misidentified lines (See ICLAC register)	No commonly misidentified cell lines were used

Human research participants

Policy information about studies involving human research participants

Population characteristics	CD34+ cells were isolated from five healthy donors (3 female, 2 male) with ages of 26, 45, 53, 58 and 62.<br>Leukemic blast cells were isolated from the bone marrow of five AML patients (2 female, 3 male) with ages of 35, 58, 58, 71 and 81.
Recruitment	Samples from healthy donors are from patients who have undergone hip replacement and the bone marrow was extracted from the removed hip bones. Healthy donors were not specifically recruited but rather the samples used that were available at that moment. Samples from AML patients were extracted in their first appointment as part of the routine diagnosis protocol. AML patients were not specifically recruited but only samples from patients used that had very high leukemic blast counts (>75), and thus presumably a more aggressive disease.
Ethics oversight	Healthy donors: Ethical committee, Faculty of Medicine, Tecnical University of Munich (TUM-MED), Germany<br>Leukemic blast cells: Ethical committee, Hospital Germans Trias i Pujol, Badalona, Spain

Note that full information on the approval of the study protocol must also be provided in the manuscript.

Flow Cytometry

Plots

Confirm that:

- [x] The axis labels state the marker and fluorochrome used (e.g. CD4-FITC).
- [x] The axis scales are clearly visible. Include numbers along axes only for bottom left plot of group (a 'group' is an analysis of identical markers).
- [x] All plots are contour plots with outliers or pseudocolor plots.
- [x] A numerical value for number of cells or percentage (with statistics) is provided.

Methodology

Sample preparation	To determine cell viability and apoptosis in the described cell lines, cells were treated for four days with the indicated inhibitors. Cell viability was then assessed by staining with 1µg/mL DAPI (4',6-Diamidino-2-phenylindole dihydrochloride) (Thermo Scientific), and 100µM MitoTracker® Red CMXRos (Thermo Scientific), while apoptotic cells were determined by staining with AnnexinV-APC (BD Biosciences, 1:200 dilution) and 1µg/mL DAPI.<br>For bone marrow samples from AML patients, only samples from patients with high blast count (>75) were used. Samples were thawed, seeded on top of HS-5 stroma cells and a few hours later treated with different concentrations of AZA. One day later, cells were treated with different concentrations of A-485, CCS1477 or DMSO for two additional days. The number of apoptotic cells was determined using AnnexinV-APC as described above. To exclude the signal from co-cultured HS-5 cells, samples were co-stained with Pharmingen™ PE Mouse Anti-Human CD90 Clone: 5E10 (BD Biosciences, Cat: 561970, 1:400 dilution) and only the CD90-negative fraction used for further analysis.<br>For protein synthesis analysis, CD34+ cells were isolated from healthy donors with immunomagnetic beads (Miltenyi Biotec, Bergisch Gladbach, Germany) and cultured for 48h. CD34+ cells were washed and resuspended in media with different concentrations of C646 (Sigma-Aldrich) and incubated for another 8 hours after which protein synthesis was analyzed with the Protein Synthesis Assay Kit Red (Abcam) according to the manufacturer's instructions. Leukemic blast cells or SKK-1 and MOLM-13 cells were treated with different concentrations of AZA, C646, A-485, CCS1477 or DMSO and incubated for 3 or 6 hours, respectively. Cells treated 1.5 hours with cycloheximide (CHX) were used as positive control. The samples were then processed using the Click-iT™ HPG Alexa Fluor™ 594 kit (Thermo Scientific) according to the manufacturer's instructions.
Instrument	LSR Fortessa cytometer and Beckman Coulter Cyan DP, Beckman Coulter
Software	BD FACSDivaTM software, FlowJo v10, FlowLogic v8
Cell population abundance	Sorting was not performed

Gating strategy

The gating strategy included FSC/SSC for cell morphology and FSC-A/FSC-H for single cells. To determine cell viability, MitoTracker® Red CMXRos (G610-A) stained cells as a live cell marker while DAPI (V450-A) stained dead cells. To determine apoptotic cells, cells stained with AnnexinV-APC (R660-A) were considered early apoptotic and cells stained with both AnnexinV-APC and DAPI (V450-A) considered late apoptotic. Co-cultured HS-5 stroma cells were excluded by staining with Pharmingen™ PE Mouse Anti-Human CD90 (BD Biosciences.

☒ Tick this box to confirm that a figure exemplifying the gating strategy is provided in the Supplementary Information.
